# COVID-19 Fear, Health Behaviors, and Subjective Health Status of Call Center Workers

**DOI:** 10.3390/ijerph19159005

**Published:** 2022-07-24

**Authors:** Hye-Ryoung Kim, Hwa-Mi Yang

**Affiliations:** 1 College of Nursing, ShinHan University, Dongducheon-si 11340, Korea; hrkim@shinhan.ac.kr; 2 Department of Nursing, Daejin University, Pocheon-si 11159, Korea

**Keywords:** COVID-19, health behavior, fear, call center, stress

## Abstract

Background: Fear may be critical in explaining individual and social behaviors. This study investigates the association between COVID-19 fear and health behavior and subjective health status changes of call center workers in the COVID-19 era. Methods: This cross-sectional study uses an online survey with 339 call center workers. We measured COVID-19 fear, health behaviors, and subjective health, and analyzed with the Macnemar or paired *t*-test, ANOVA or χ^2^ test, Scheffe’s test, and multiple linear regression. Results: COVID-19 fear was associated with poor stress management, shorter sleep hours, and binge eating. Moreover, COVID-19 fear and time pressure at work were negatively associated with subjective health status. Conclusion: Strengthening the support system for call center workers to manage the COVID-19 fear might be essential. Moreover, there is a need to improve dense environments and reduce time pressure by ensuring adequate rest time and increasing physical activity.

## 1. Introduction

The COVID-19 pandemic not only poses safety, work efficiency, and psychosocial risks that play an essential role in occupational health, but the continuation of the pandemic may increase work responsibilities with a sense of crisis for economic deterioration [1]. The COVID-19 outbreak can cause emotional distress and anxiety, exacerbating existing mental health problems and forming stress-related disorders in those affected [2]. There is evidence that the COVID-19 pandemic harms psychological well-being [3,4,5]. In a study in China, a significant proportion of subjects reported depression, anxiety, and psychological stress symptoms, and more than 50% of the public rated COVID-19-related psychological effects as moderate or severe [5]. A Cross-Country Examination on the Fear of COVID-19 study found that COVID-19 fear was greater among women and older groups [4].

A call center is where companies and customers communicate through information and communication means, and female workers form the majority [6]. Call center work is primarily divided into inbound, outbound call service, and inbound/outbound call blending work. The inbound task is to handle customer needs and complaints, order receipt processing, product description, confirm, and assure the customer’s questions or queries. In contrast, the outbound task is responsible for active sales and marketing, campaign development, et cetera. Call center employees inevitably experience much stress and emotional exhaustion [7]. In addition, a work environment in which several people are in charge of phone work in a narrow office is inevitably vulnerable to transmission of infection [6]. The long working hours of call center employees in densely populated spaces might have resulted in large-scale cluster infections of COVID-19 in Korea. The fear of COVID-19 infection among call center workers would increase in such a situation.

Regarding the COVID-19 pandemic, fear may be critical in explaining individual and social behaviors [8]. Fear-linked risk perception plays a direct and indirect role in preventive behaviors [8]. As such, COVID-19 fear can affect health behaviors. COVID-19 fear mediates the relationship between perceived risk and preventive health behaviors [9]. Some studies have reported changes in regular eating patterns such as skipping meals [10], reducing eating out [11], increasing sedentary behavior, and decreasing physical activity due to the effects of COVID-19 fear and anxiety on health behavior [11].

People exposed to high-stress levels experience further poor health than those with low-stress levels [12]. When a worker’s stress is unbearable or continuously experienced, a negative self-concept might be formed, leading to unhealthy behaviors such as drinking, smoking, and drug abuse. The job demands, stresses, and flawed work environment associated with communicating and providing services directly to people can affect burnout for call center workers [12].

This study investigates the association between COVID-19 fear and health behavior and subjective health status changes of call center workers in the COVID-19 era. The questions of this study are as follows: 1. Is there a difference in health behaviors and subjective health status before and after the COVID-19 pandemic? 2. Is there a difference in health behavior and subjective health status according to the degree of fear of COVID-19? 3. What factors are related to each health behavior and subjective health status? The results of this study will be the basis for developing interventions to improve the health behavior of call center workers who are vulnerable to occupational health. 

## 2. Methods

### 2.1. Study Design and Participants

This cross-sectional study uses an online survey with 339 call center workers. We recruited participants from an outsourcing service company, which employs most call center staff in Seoul, South Korea, with more than 30 centers. Call center workers between the ages of 22 and 60, who understood the purpose of the study, agreed to participate, and responded to the online questionnaire were included. The exclusion criterion was less than two years of work experience to confirm the effect of the COVID-19 pandemic.

We calculated the sample size using G-power version 3.1 (Heinrich Heine University, Dusseldorf, Germany). Based on a multiple linear regression analysis with the effect size of health behavior change as 0.08 [13], the significance level at 0.05, test power at 0.95, the number of tested predictors as 5, and total predictor as 13; the minimum required sample size was 254. However, we recruited 339 participants for data stability.

### 2.2. Data Collection

The participant recruitment and data collection period were from 15 November 2021, to 15 December 2021. In the recruitment procedure, the researcher explained the purpose and contents of the study to the safety and health manager of the relevant workplace. After obtaining cooperation, a notice of recruitment was posted on the bulletin board of each department to recruit participants. All participants filled out electronic informed consent before the survey. Data was collected using an online survey, not allowing duplicate or missing responses. 

### 2.3. Measurements

#### 2.3.1. COVID-19 Fear

We measured the COVID-19 fear by the fear of COVID-19 scale developed by Ahorsu et al. [14]. It consists of 7 items on a 5-point Likert scale, with a minimum of 7 points and a maximum of 35 points, with higher scores indicating higher levels of fear relating to the COVID-19 pandemic. A study involving Egyptian physicians defined it as normal with 7–16 points, 17–26 points as moderate, and 27 points or more as severe fear [15]. In Ahorsu, Lin, Imani, Saffari, Griffiths, and Pakpour’s study [14], Cronbach’s alpha of the Malaysian version was 0.82. In this study, one of the researchers translated the scale into Korean. After that, the other researcher back-translated it. Then we compared the meaning. Three nursing professors and two psychiatrists checked the validity of the Korean version. The reliability in this study was 0.91.

#### 2.3.2. Health Behaviors

We measured pre-pandemic and current smoking status, high-risk drinking, moderate-intensity physical activity, stress management, binge eating, sedentary behavior, and sleep time as health behaviors. A single item asks for each category in Korean. According to the Korean national guideline of hazardous drinking, we defined high-risk drinking as seven or more drinks for men and five or more drinks for women at a time [16]. For moderate-intensity physical activity: “How many days per week do you usually engage in moderate-intensity exercise that increases your heart rate for more than 30 min and makes you breathe harder than usual? (Example: walking fast, carrying light luggage)” was asked, and we classified as having physical activity in moderate-intensity for more than five days [17]. For stress management measurement, we asked, “Do you use positive methods (meditation, music, movies, travel, et cetera.) to relieve tension and pressure in your way when you are stressed?” Responses were on a Likert scale ranging from 1 to 5; a higher score means better stress management. The occurrence of binge eating was asked by, “How many days a week do you overeat or binge eat due to stress?” scored by 0–7, and the higher the score, the higher the binge-eating behavior. Moreover, we asked for sedentary hours and sleep hours per day. 

We asked all questions by dividing the pre-pandemic and current sections in parallel. Every response on pre-pandemic was based on the participant’s recollection.

#### 2.3.3. Time Pressure at Work

Time pressure at work, composed of 4 items on a 5-point Likert scale, was measured to check the work stress of call center workers who have to handle work within a specific time efficiently (e.g., “My work is always time-pressed”). The reliability indicated by Cronbach’s alpha in the previous study was 0.72 to 0.77 [18], and 0.87 in this study.

#### 2.3.4. Subjective Health Status

We asked about the subjective health status on a 100-point scale: “If you were to score your health status on a 100-point scale, how many points would it be (pre-pandemic and current state)?” The higher the score, the higher the subjective health status.

### 2.4. Data Analysis

We analyzed all data statistically using the SPSS/WIN 23.0 program (IBM, Armonk, NY, USA). We presented demographic characteristics of participants with descriptive statistics of frequency, percentage, mean, standard deviation, and range. The Macnemar test or paired *t*-test analyzed the difference between health behaviors between pre-pandemic and current. ANOVA test or χ^2^ test compares differences in health behavior according to COVID-19 fear level (mild, moderate, and severe group), and Scheffe’s test confirmed the post-hoc comparison. We adopted multiple linear regression to confirm the association between COVID-19 fear (total score) of call center workers and health behavior or subjective health status in the total variance with demographics and working condition variables. For multiple linear regression, we designated the participant’s general characteristics as covariates, fear of COVID-19 as the main variables, and simultaneously input each of the four health behaviors and subjective health status as dependent variables.

### 2.5. Ethical Considerations

The Institutional Review Board of Daejin University approved the research methodology and procedures related to ethical concerns (IRB number: 1040656-202110-HR-01-17).

## 3. Results

### 3.1. Demographic Characteristics of Participants

Female participants account for 90.3%. The average age of the participants was 43.9 years, and the age range was 22–60 years. Most participants were full-time workers (95.3%). Inbound service and home-based workers account for 56.6 and 35.4%. The average score of the time pressure at work was 3.11, and the total score of the COVID-19 fear was 19.8 out of 35 (Table 1).

### 3.2. Health Behavior and Subjective Health Status According to the COVID-19 Era

There were no differences between pre-COVID-19 and present smoking, high-risk drinking, and moderate-intensity physical activity (Table 2). However, compared to pre-COVID-19, the current stress management was poor (*t* = 4.43, *p* < 0.001), binge eating (*t* = −4.54, *p* < 0.001), and sedentary behavior (*t* = −5.40, *p* < 0.001) increased, and sleep hours (*t* = 3.16, *p* = 0.002) decreased. The average score of subjective health status was 70.22 out of 100 points pre-COVID-19, which was higher than the current 65.74 points (*t* = 6.29, *p* < 0.001).

### 3.3. Health Behavior and Subjective Health Status, According to Fear of COVID-19

Two hundred and fifty-two workers (74.3%) felt more than moderate COVID-19 fear; among them, 37 workers felt severe fear. In the severe fear of COVID-19 group, the stress management score (F = 4.39, *p* = 0.013) was the lowest, while the binge eating score (F = 11.78, *p* < 0.001) was the highest. Sleep hours (F = 4.77, *p* = 0.009) was the longest in the normal group, and subjective health status (F = 5.86, *p* = 0.003) was the lowest in the severe group (Table 3).

### 3.4. Factors for Health Behavior and Subjective Health Status

Fear of COVID-19 had a negative association with stress management (β = −0.15, *p* = 0.007) and sleep hours (β = −0.12, *p* = 0.035), whereas a positive association with binge eating (β = 0.23, *p* < 0.001). Home-based working positively related to sleep hours (β = 0.23, *p* < 0.001). Fear of COVID-19 (β = −0.15, *p* = 0.006) and time pressure at work (β = −0.11, *p* = 0.049) were negatively associated with subjective health status (Table 4).

## 4. Discussion

The COVID-19 pandemic affects occupational health by causing immediate infection problems, stress, anxiety, and fear. In this study of call center workers, the participants’ fear of COVID-19 was 19.8 out of 35, slightly higher than the median. This fear level is high compared to 15.9–17.8 in multiple sclerosis patients [13], and 19.9 in nurses at the forefront of COVID-19 [19]. The concern and anxiety about infection must have been profound, experiencing group infection outbreaks due to the work environment where several people work together in a narrow space.

There were no differences between the pre and current pandemic in smoking and high-risk drinking cases. However, some studies have reported increased smoking and drinking during the pandemic [20,21,22]. These results may differ because of the measurement. This study measured whether call center workers were smoking, or high-risk drinking or not, whereas other studies directly investigated the amount of drinking and smoking. Moreover, most of the participants in this study were women, and the Korean culture has an unfriendly perception of women smoking and drinking. In this regard, the small proportion of smokers or high-risk drinking women also had an effect. These suggest that although non-smokers do not start smoking anew during the pandemic, existing smokers may increase their smoking. Furthermore, alcohol consumption may increase even if it is not high-risk drinking.

Participants in this study reported changes in their health behaviors such as stress management, diet habit, and sleeping hours between the pre-COVID-19 pandemic and current times based on their recollections. In the case of stress management, positive coping has decreased. These results correspond to a study comparing the beginning of and six months into the pandemic [20]. Under the influence of the pandemic, social distancing, lockdown, and reduced social contact induce high stress in people. As this situation continues, maladaptive stress-coping may prevail over positive stress-coping. In the case of call center employees working in a poor environment, with an increased workload dealing with customers exhausted by COVID-19, concerns, and fears of infection aggravate the stressful situation, making it difficult to cope with stress without an adequate supporting system. According to Lazarus & Folkman’s theory of stress coping, primary appraisal evaluates how dangerous it is when recognizing stress. The secondary appraisal assesses the possibility and resources for stress management. If considered coping resources are sufficient, a positive response may increase; otherwise, a maladaptive response may ensue [23]. Thus, when the support system is broken due to increased uncertainty and lack of social contact during the pandemic, maladaptive coping such as denial, use of psychoactive substances, suppression of activities, and self-blame increases [20]. Therefore, there is a need to strengthen the support system for call center workers’ psychological health in the COVID-19 pandemic.

Anxiety and fear from COVID-19 may affect dietary habits and food preferences [10]. This study also confirms an increase in binge eating compared to pre-pandemic. These results support the findings of an Italian study on pre-existing binge eating disorders exacerbated during COVID-19 lockdown [24]. Higher stresses related to the pandemic and awareness of weakened support systems may increase binge eating behavior.

The research literature has highlighted the increased prevalence of sleep disorders across different countries, including poor sleep quality through COVID-19 [25]. Depression, anxiety, and stress symptoms may affect the sleep pattern. In this study, sleep duration decreased by about 14 minutes during the pandemic compared to an average decrease of 38 minutes in a study with healthcare workers [26]. Still, it is necessary to confirm whether the decreased sleep duration is a problem with the onset or duration of sleep. It was also confirmed in this study, that home-based work positively affected sleep hours. We infer that there was a relieving effect on pandemic fear away from the dense working environment of the call center, and as a result, it positively impacted sleep hours. Therefore, it will be necessary to improve the dense working environment for the occupational health of call center workers.

Many studies have reported increased sedentary behavior and decreased physical activity due to COVID-19 lockdown [20,26,27,28,29]. However, although sedentary behavior increased during the pandemic in this study, it was difficult to confirm the decrease in physical activity. This finding may result from measuring the proportion of participants who met the recommended moderate or higher intensity physical activity. There also may be an effect that the proportion of participants who met the recommended amount was low. Regardless of COVID-19, it is necessary to develop a strategy to increase the physical activity of call center workers in terms of occupational health.

In this study, subjective health status was lower in current times than before the pandemic, and the group with high COVID-19 fear reported lower subjective health status. Moreover, as a result of multiple regression analyses, it was confirmed that time pressure at work and COVID-19 fear were negatively associated. Time pressure at work affects perceptions of occupational stress, negatively affecting subjective perceptions of health, consequently lowering work ability [30]. A study of 488 employees working in a Dutch telecommunication company call center also reported that work pressure, such as time pressure as an occupational demand, was the most important predictor of health problems [31]. Therefore, it is necessary to create an environment to reduce time pressure by securing appropriate break times for call center employees.

There are some limitations to this study. First, there is a risk of recall bias regarding the simultaneous measurement of variables before and after the pandemic outbreak. Moreover, single items in each category measure health. There needs to be caution related to integrated interpretation. Second, the participants were primarily female. However, this was inevitable due to the characteristics of call center work. We propose further studies of workers in various occupational groups. Third, we could not consider the cultural differences for the group classification according to COVID-19 fear level because the COVID-19 fear scale has been developed relatively recently and lacks data from various population groups. Moreover, this is the first study adopting the scale for the Korean population. Despite these limitations, this study is meaningful in that it laid the basis for developing interventions to improve the occupational health of call center workers in the context of COVID-19.

## 5. Conclusions

We investigated the COVID-19 fear among call center workers and the association between COVID-19 fear and health behavior and the subjective health status in the COVID-19 era. The study showed the association between COVID-19 fear and poor stress management, shorter sleep hours, binge eating, and poor subjective health status. COVID-19 fear can affect health behaviors. Therefore, preparing a plan to manage COVID-19 fear is necessary for occupational health. Strengthening the support system for call center workers’ psychological health might be a solution. 

## Figures and Tables

**Table 1:** General Characteristics of Participants (N = 339).

Variables	N (%) or Mean ± SD	Range
Age (year)	43.9 ± 7.53	22.0–60.0
Gender		
Female	306 (90.3)	
Male	33 (9.7)	
Education (≥College)	184 (54.3)	
Marital status		
Married	185 (54.6)	
Unmarried/separated/divorced	154 (45.4)	
Number of children	1.0 ± 0.96	0.0–3.0
Monthly household income (≥4 million KRW)	163 (48.1)	
Perceived socioeconomic level (1–5)	2.6 ± 0.61	1.0–4.0
Years of current work experience	5.9 ± 3.45	2.0–34.0
Type of employment		
Full-time worker	323 (95.3)	
Part-time worker	16 (4.7)	
Occupational category		
Counselor	275 (81.1)	
Team or center leader	51 (15.1)	
Support staff	13 (3.8)	
Counseling service characteristics		
Inbound	192 (56.6)	
Outbound	99 (29.2)	
Call blending	7 (2.1)	
Business support	41 (12.1)	
Home-based working	120 (35.4)	
Office-based working	219 (64.6)	
Working hours per week	33.6 ± 14.48	4.0–70.0
Working hours per week (>40 h)	88 (26.0)	
Time-pressure at work	3.1 ± 0.80	1.0–5.0
Degree of fear of COVID-19 (total score)	19.8 ± 5.43	7.0–35.0
Normal	87 (25.7)	
Moderate	215 (63.4)	
Severe	37 (10.9)	

SD = standard deviation; KRW = Korean won.

**Table 2:** Comparison of Health Behaviors pre and present COVID-19.

Variables	N (%) or Mean ± SD		*t*/χ^2^	*p*
	Pre-COVID 19	Present		
Current smoking (yes)	77 (22.7)	77 (22.7)	339.00	1.000
High-risk alcohol drinking	79 (23.3)	79 (23.3)	282.17	1.000
Moderate PA (≥5 days/week)	22 (6.5)	17 (5.0)	169.74	0.227
Stress management (1–5 scores)	2.9 ± 0.99	2.7 ± 0.98	4.43	<0.001
Binge eating (0–7 scores)	1.2 ± 1.21	1.4 ± 1.50	−4.54	<0.001
Sedentary behavior hours/day	8.6 ± 5.31	9.0 ± 5.35	−5.40	<0.001
Sleep hours/day	6.3 ± 1.36	6.1 ± 1.34	3.16	0.002
Subjective health status (0–100 scores)	70.6 ± 15.87	65.9 ± 15.79	5.68	<0.001

SD = standard deviation; PA = physical activity; *t* = paired *t*-test; χ^2^ = macnemar.

**Table 3:** Health Behavior and Subjective Health, According to Fear of COVID-19.

Variables	Normal ^a^ (*n* = 87)	Moderate ^b^ (*n* = 215)	Severe ^c^ (*n* = 37)	F/χ^2^	*p*	Scheffe’s Test
	N (%) or Mean ± SD					
Current smoking (yes)	27 (31.0)	41 (19.1)	9 (24.3)	5.112	0.078	
High-risk alcohol drinking	22 (25.3)	43 (20.0)	14 (37.8)	5.877	0.054	
Moderate PA (≥5 days/week)	5 (5.7)	12 (5.6)	0 (0.0)	2.196	0.333	
Stress management (1–5 scores)	2.8 ± 1.02	2.8 ± 0.95	2.3 ± 0.97	4.394	0.013	c < a = b
Binge eating (0–7 scores)	1.3 ± 1.44	1.3 ± 1.39	2.5 ± 1.82	11.776	<0.001	a = b < c
Sedentary behavior hours/day	9.4 ± 5.53	9.0 ± 5.39	8.3 ± 4.71	0.522	0.594	
Sleep hours/day	6.5 ± 1.92	6.1 ± 1.03	5.7 ± 1.10	4.773	0.009	b = c < a
Subjective health status (0–100 scores)	67.9 ± 17.74	66.5 ± 15.09	57.8 ± 17.82	5.861	0.003	c < a = b

Scheffe’s test confirmed the post-hoc comparison; Level of COVID-19 fear: ^a^ Normal (7–16 points), ^b^ Moderate (17–26 points), ^c^ Severe (27 points or more).

**Table 4:** Factors for Health Behavior and Subjective Health.

Variables	Stress Management (1–5 Scores)	Binge Eating (0–7 Scores)	Sedentary Behavior Hour/Day	Sleep Hours/Day	Subjective Health Status (0–100 Scores)
	β (SE)				
Age	0.054 (0.009)	−0.289 (0.012) **	−0.049 (0.048)	−0.020 (0.012)	0.209 (0.438) **
Gender (men)	−0.039 (0.187)	−0.142 (0.266) **	0.068 (1.026)	0.089 (0.252)	0.031 (2.955)
Education (≥College)	0.089 (0.109)	0.027 (0.156)	0.085 (0.601)	0.029 (0.147)	0.043 (1.730)
Marital status (married)	0.139 (0.154)	0.119 (0.219)	−0.129 (0.844)	−0.141 (0.207)	−0.031 (2.429)
Number of children	−0.240 (0.086) **	−0.098 (0.113)	−0.018 (0.474)	0.117 (0.116)	−0.009 (1.365)
Years of current work experience	0.018 (0.016)	0.004 (0.023)	−0.022 (0.090)	0.169 (0.022) **	−0.055 (0.260)
Type of employment (regular)	−0.027 (0.253)	0.029 (0.360)	0.010 (1.389)	−0.070 (0.341)	0.022 (3.998)
Occupational Category (counselor)	0.039 (0.147)	0.017 (0.210)	−0.032 (0.810)	0.053 (0.199)	−0.100 (2.332)
Counseling service (inbound)	−0.089 (0.121)	0.094 (0.172)	−0.001 (0.664)	−0.017 (0.163)	−0.094 (1.911)
Home-based working	−0.020 (0.118)	−0.002 (0.168)	−0.030 (0.650)	0.128 (0.159) *	0.060 (1.871)
Working hours per week (>40 h)	0.025 (0.129)	0.008 (0.183)	0.083 (0.707)	0.026 (0.174)	0.002 (2.037)
Time-pressure at work	−0.072 (0.068)	0.053 (0.096)	0.089 (0.371)	0.003 (0.091)	−0.107 (1.069) *
Fear of COVID-19	−0.151 (0.010) **	0.227 (0.014) **	−0.038 (0.055)	−0.116 (0.014) *	−0.151 (0.159) **
R-square	0.07	0.20	0.06	0.10	0.11

β = standardized beta, SE = standard error, * *p* < 0.05. ** *p* < 0.01.

## Data Availability

The data supporting this study’s findings are available from the corresponding author, (H-M, Yang), upon reasonable request.
